# Predictability of Evolutionary Trajectories in Fitness Landscapes

**DOI:** 10.1371/journal.pcbi.1002302

**Published:** 2011-12-15

**Authors:** Alexander E. Lobkovsky, Yuri I. Wolf, Eugene V. Koonin

**Affiliations:** National Center for Biotechnology Information, National Library of Medicine, National Institutes of Health, Bethesda, Maryland, United States of America; Harvard University, United States of America

## Abstract

Experimental studies on enzyme evolution show that only a small fraction of all possible mutation trajectories are accessible to evolution. However, these experiments deal with individual enzymes and explore a tiny part of the fitness landscape. We report an exhaustive analysis of fitness landscapes constructed with an off-lattice model of protein folding where fitness is equated with robustness to misfolding. This model mimics the essential features of the interactions between amino acids, is consistent with the key paradigms of protein folding and reproduces the universal distribution of evolutionary rates among orthologous proteins. We introduce mean path divergence as a quantitative measure of the degree to which the starting and ending points determine the path of evolution in fitness landscapes. Global measures of landscape roughness are good predictors of path divergence in all studied landscapes: the mean path divergence is greater in smooth landscapes than in rough ones. The model-derived and experimental landscapes are significantly smoother than random landscapes and resemble additive landscapes perturbed with moderate amounts of noise; thus, these landscapes are substantially robust to mutation. The model landscapes show a deficit of suboptimal peaks even compared with noisy additive landscapes with similar overall roughness. We suggest that smoothness and the substantial deficit of peaks in the fitness landscapes of protein evolution are fundamental consequences of the physics of protein folding.

## Author Summary

Is evolution deterministic, hence predictable, or stochastic, that is unpredictable? What would happen if one could “replay the tape of evolution”: will the outcomes of evolution be completely different or is evolution so constrained that history will be repeated? Arguably, these questions are among the most intriguing and most difficult in evolutionary biology. In other words, the predictability of evolution depends on the fraction of the trajectories on fitness landscapes that are accessible for evolutionary exploration. Because direct experimental investigation of fitness landscapes is technically challenging, the available studies only explore a minuscule portion of the landscape for individual enzymes. We therefore sought to investigate the topography of fitness landscapes within the framework of a previously developed model of protein folding and evolution where fitness is equated with robustness to misfolding. We show that model-derived and experimental landscapes are significantly smoother than random landscapes and resemble moderately perturbed additive landscapes; thus, these landscapes are substantially robust to mutation. The model landscapes show a deficit of suboptimal peaks even compared with noisy additive landscapes with similar overall roughness. Thus, the smoothness and substantial deficit of peaks in fitness landscapes of protein evolution could be fundamental consequences of the physics of protein folding.

## Introduction

One of the most intriguing questions in evolutionary biology is: to what extent evolution is deterministic and to what extent it is stochastic and hence unpredictable? In other words, what happens if “the tape of evolution is replayed:” are we going to see completely different outcomes or the constraints are so strong that history will be repeated [1]–[4]? If evolution is envisaged as movement of a population across a fitness landscape, the question can be reworded more specifically: among the numerous trajectories connecting any two points on the landscape, what fraction is accessible to evolution? Until recently, these remained purely theoretical questions as experimental study of fitness landscapes in the actual sequence space was impractical, due both to the technical difficulty of producing and assaying numerous expressed sequence variants and to the more fundamental problem of defining an adequate quantitative measure of fitness. However, recent experimental studies of fitness landscapes could potentially shed light on the problem of evolutionary path predictability.

The most thoroughly characterized feature of empirical fitness landscapes is the structure near a peak. In experiments that examine the peak structure, a high fitness sequence is typically subjected to either random mutations or an exhaustive set of mutations at a small number of important sites. The resulting library of mutants is then assayed to measure a proxy of fitness [5]–[9]. Significant sign epistasis (a situation in which the fitness effect of a particular mutation can be either positive or negative depending on the genetic context) has been observed. Deviations from the additive fitness model have been found to be independent of the genetic context and purely random [10]–[13]. Because these studies characterize only a small region of the landscape, they cannot be used to address the question of path predictability.

Another broad class of experiments probes the evolutionary trajectories from low to high fitness. Usually, in such experiments, a random peptide is subjected to repeated rounds of random mutagenesis and purifying selection [8], [14]–[17]. During this process fitness grows with each generation and eventually stagnates at a suboptimal plateau. The characteristics of the fitness growth as well as the dependence of the plateau height on the library size can be used to classify landscapes [18]. A quantitative comparison to the 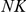 model of random epistatic landscapes (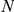 is the number of sites in an evolving sequence and 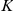 is the number of sites that affect the fitness contribution of a particular site through epistatic interactions) can even yield quantitative estimates of 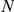 and 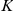 [19], [20]. The directed evolution studies explore the evolutionarily accessible portion of the landscape and could in principle be used to shed light on the question of path predictability. However, the inaccessible regions of the landscape remain unexplored and the volume of data at this point is insufficient to obtain quantitative conclusions regarding path predictability.

A different type of landscapes has been explored in various microarray experiments where protein-DNA(RNA) binding affinity serves as the proxy for fitness [21], [22]. These experiments produce vast, densely sampled landscapes. A comparison with a sophisticated Landscape State Machine model of a correlated fitness landscapes yields estimates of the model parameters [23], [24]. The DNA binding landscapes, in principle, contain the information required for the analysis of path statistics, and could be a valuable resource for advancing the understanding of evolutionary path predictability.

Empirical studies that exhaustively sample a region of the fitness landscape allow one to actually assess the accessibility of the entire set of theoretically possible evolutionary trajectories in a particular (small) area of the fitness landscape. For example, all mutational paths between two states of an enzyme, e.g., the transition from an antibiotic-sensitive to an antibiotic resistant form of 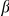-lactamase [25]–[27] or the transition between different specificities of sesquiterpene synthase [28] have been explored. The results of these experiments, which out of necessity explore only short mutational paths of 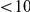 amino acid replacements, suggest that there is a substantial deterministic component to protein evolution: only a small fraction of the possible paths are accessible for evolution [25], [29]–[31].

Recent analyses of fitness data have revealed dense networks of genetic and molecular interactions responsible for the substantial ruggedness and sign epistasis of empirical fitness landscapes [13], [32]. The emerging quantitative analysis of fitness landscapes can shed light on some of the most fundamental aspects of evolution but the interpretation of the currently available experimental results requires utmost caution as only a minuscule part of the sequence space can be explored, and that only for a few more or less arbitrarily selected experimental systems.

Here we focus on the question of the predictability of mutational paths which is intimately tied to the ruggedness/smoothness of the fitness landscapes. The study of random landscapes of low dimensionality revealed an intuitively plausible negative correlation between the roughness of a landscape and the availability of pathways of monotonic fitness [33]. In the same study, Carneiro and Hartl showed that experimentally characterized landscapes are significantly smoother than their permuted counterparts and exhibit greater peak accessibility [33].

To gain insights into the structure of the fitness landscapes of protein evolution and in particular the accessibility of mutational paths we used a previously developed simple model of protein folding and evolution [34]. The key assumption of this model, which is based on the concept of misfolding-driven evolution of proteins [35]–[37], is that the fitness of model proteins is determined solely by the number of misfolded copies that are produced before the required abundance of the correctly folded protein is reached. We have previously shown that this model accurately reproduces the shape of the universal distribution of the evolutionary rates among orthologous protein-coding genes along with the dependencies of the evolutionary rate on protein abundance and effective population size [34]. These results appear to suggest that our folding model (described in detail the Methods section) is sufficiently rich to reproduce some of the salient aspects of evolution. The model is also simple enough to allow exhaustive exploration of the fitness landscapes, which prompted us to directly address the problem of evolutionary path predictability.

We build on the efforts of Carneiro and Hartl [33] who examined the statistics of evolutionary trajectories. Although counting monotonic fitness paths reveals important features of the landscapes, we argue that reliable retrodiction of the evolutionary past is possible (i.e., evolution is quasi-deterministic) only when the available monotonic paths are similar to each other in a quantifiable way. We therefore propose a measure of path divergence to quantify the difference between the available monotonic paths. Our aims are to investigate the structure of the fitness landscapes of protein evolution and to elucidate the connection between the roughness of landscapes and the predictability of mutational trajectories. We analyze three classes of fitness landscapes: landscapes in which fitness is derived from the folding robustness of model polymers; additive random landscapes perturbed by noise; and experimental landscapes derived from the combinatorial mutation analysis of drug resistance and enzymatic activity. We show that all three classes of landscapes are markedly smoother than their randomly permuted counterparts and all exhibit a similar qualitative connection between roughness and path predictability. However, at the same level of path predictability, the folding landscapes have substantially fewer fitness peaks. Equivalently, mutation paths are more predictable than one would expect based on the number of peaks if the landscapes were uncorrelated. Given that the statistical properties of the model landscapes can be directly traced to the constraints imposed by the energetics and kinetics of a folding heteropolymer, we hypothesize that the relative smoothness and the suppression of suboptimal peaks in fitness landscapes of protein evolution are fundamental consequences of protein folding physics.

## Results

### Quantitative characterization of fitness landscapes

Carneiro and Hartl compared small random landscapes to several empirical fitness landscapes using deviation from additivity as a measure of roughness [33]. They found that empirical landscapes were significantly smoother than their random counterparts and that the degree of smoothness was correlated with the number of monotonic paths to the main summit. Deviation from additivity of a landscape is computed by fitting an additive model in which the fitness of each sequence is different from the peak fitness by the sum of contributions of the substitutions that differentiate it from the peak sequence. The negative fitness contributions of the substitutions to the peak fitness are adjusted to minimize the sum 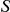 of squares of the differences between the actual fitnesses in the landscape and the fitnesses predicted by the additive model. Deviation from additivity is defined as 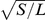, where 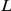 is the number of points in the landscape.

**Figure 1:**
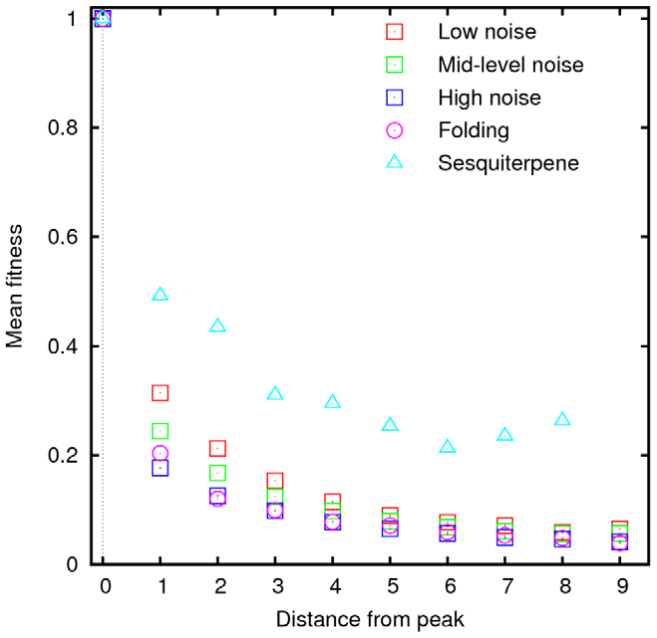
Fitness averaged over all points at a particular distance 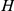 from the peak for folding landscapes, additive landscapes with the same three levels of multiplicative noise used in Fig. 6 and the sesquiterpene synthase landscape.

Because roughness of a multidimensional landscape with variable degree connectivity is not an intuitive concept, we introduce three additional quantitative measures to probe alternative facets of the concept of roughness. First, local roughness is the root mean squared difference between the fitness of a point and its neighbors, averaged over the entire landscape. As defined, local roughness conflates the measures of roughness and “steepness.” For example, a globally smooth landscape, in which fitness depends only on the distance from the peak, will have a non-zero local roughness. However, because there is a large number of directions that change the distance from the peak by one, the local roughness of a globally smooth landscape will be vanishingly small. In addition, our landscapes tend to be globally flat–so that the average decrease in fitness due to a single mutation step away from the main peak is much smaller than the local fitness variability–everywhere except a small region around the main peak (see Fig. 1). Therefore, the landscape-average local roughness in our case is a true measure of the local fitness variability.

Second, the fraction of peaks is the number of points with no fitter neighbors divided by the total number of points in the landscape. A strictly additive landscape has a single peak [30] whereas the peak fraction in landscapes derived from the folding model as well as the corresponding randomized landscapes depends on the method of landscape construction, alphabet size and sequence length.

Third, the roughness of a landscape can be assessed by identifying its tree component. The tree component is the set of all nodes with no more than one neighbor of higher fitness. Thus, the tree component includes peaks and plateaus. Monotonic fitness paths along the tree component form a single or several disjoint tree structures without loops. In the limit of high selection pressure, a mutational trajectory that finds itself on the tree component has a single path to the nearest peak or plateau, i.e. evolution on the tree component is completely deterministic. We use the mean distance to the tree component, i.e. the distance to the tree component averaged over the landscape, as a measure of roughness. In a fully additive landscape, only the peak sequence and its immediate neighbors belong to the tree component and therefore the mean distance to the tree component is a measure of the diameter of an additive landscape (which, for example, could be defined as the maximum pairwise distance between points on the landscape). Kauffman and Levin have shown that in a large class of correlated random landscapes, the mean distance to the tree component grows only logarithmically with the number of points in the landscape [19].

We utilize two quantitative measures of the predictability of evolutionary trajectories. First is fraction of monotonic paths to the main peak 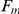 which is computed by counting the number 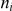 of simple (without reverse substitutions or multiple substitutions at the same site) monotonic paths to the main peak from each point 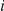 on the landscape, dividing it by the total number of simple paths 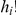 (where 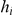 is the Hamming distance from point 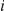 to the peak), and averaging over the landscape via
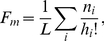
(1)where 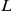 is the number of points in the landscape and the sum excludes the main peak. The monotonic path fraction 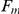 measures the scarcity of accessible evolutionary paths when selection is strong. When the monotonic path fraction is small, evolution is more constrained.

Second, the mean path divergence, is a fine-grained measure of evolutionary (un)predictability. We first define the divergence 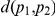 of a pair of paths 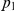 and 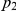, as the average of the shortest Hamming distances from each point on one path to the other path. Suppose that we have a way of generating stochastic evolutionary paths. The outcome of a large number of evolutionary dynamics simulations is a collection of paths with their associated probabilities of occurrence. In general, the probability of occurrence of an evolutionary path is proportional to the product of fixation probabilities of its constituent mutation steps. Given a bundle of paths with the same starting and ending points, we define its mean path divergence to be
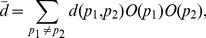
(2)where 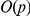 is the probability of occurrence of path 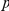 in the ensemble. In other words, if two paths were drawn from the bundle at random with probabilities proportional to 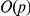, their expected divergence would be 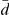. Alternatively, if we were to fix one path to be the most likely path in the bundle and to select the second path at random with probability proportional to 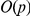, the divergence would be proportional to 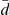 as well.

The six quantitative characteristics of fitness landscapes are summarized in Table 1.

**Table 1:** Summary of the quantitative landscape characteristics.

Name of characteristic	Characterized property	Definition
Peak fraction	Roughness	Number of points with no fitter neighbors divided by the total number of points in the landscape
Deviation from additivity	Roughness	Mean squared difference between the actual fitness and the fitness predicted by the best fit additive model scaled by the mean squared fitness in the landscape
Local roughness	Roughness	Mean squared difference between the fitness of a point and its immediate neighbors averaged over the landscape
Distance to tree component	Roughness	Shortest distance to the tree component (points with at most one uphill neighbor) averaged over the landscape
Monotonic path fraction	Path predictability	Fraction of the shortest paths (without multiple or reverse substitutions) to the main peak averaged over the landscape
Mean path divergence	Path predictability	Measure of dissimilarity (divergence) of the monotonic paths to the main peak averaged over the landscape

In an additive landscape, the mutational trajectory is maximally ambiguous. As every substitution that brings the sequence closer to the peak increases fitness, substitutions can occur in any order and all shortest mutational trajectories to the peak–without reverse substitutions or multiple substitutions at the same site–are monotonic in fitness. In the strong selection limit of our model defined below, all monotonic trajectories have roughly the same probability of occurrence, so the mutational path cannot be predicted.

The mean path divergence is a better measure of the predictability of evolutionary trajectories than the number or fraction of accessible paths. Even when only a small fraction of paths are monotonic in fitness, these paths could potentially be quite different, perhaps randomly scattered over the landscape. In such a case, prediction of the evolutionary trajectory would be inaccurate despite the scarcity of accessible paths which will be reflected in a high value of path divergence.

Equation (2) introduces the mean path divergence of a bundle of paths with the same starting and ending points. The landscape-wide mean path divergence is measured by constructing representative path bundles with all possible [start, peak] pairs including suboptimal peaks as trajectory termination points. Path divergence is averaged over all bundles with the starting and ending points separated by the same Hamming distance. To construct the path bundles, we employed a low mutation rate model in which the attempted substitutions are either eliminated or fixed in the population before the next mutation attempt occurs.

We invoke the misfolding-cost hypothesis to assign a fitness to a sequence that folds with probability 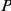 to a particular structure. To produce an abundance 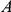 of correctly folded copies, an average of 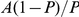 of misfolded copies are produced. The “fitness” of a sequence should be a monotonically decreasing function of the cost incurred by the misfolded proteins. Previously we showed that qualitative conclusions drawn from the average population dynamics on the fitness landscape did not depend on the precise functional relationship between the number of misfolded copies and fitness [34]. We use simply the negative of the number of misfolded copies and assign a fitness 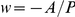, to a sequence whose probability of folding to the reference structure is 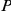. Because the exact population dynamics model is not important, we use diploid population dynamics in the low mutation rate limit. Therefore, the probability of fixation of a mutant 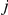 in the background of 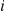 is given by
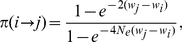
(3)where 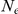 is the effective population size [38] which in all simulations was fixed at 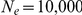. The required abundance 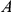 is a measure of the strength of selection. In the limit of large 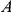, the probability of fixation of a beneficial mutation is unity whereas deleterious mutations are never fixed. Since the effective population size is large in our simulations, neutral mutations are almost never fixed either. Because uphill steps in the fitness landscape are equally likely, all monotonic uphill trajectories have equal evolutionary significance.

In the analysis that follows, we study the association between landscape roughness and path predictability for the folding landscapes and their randomized (also referred to as permuted or scrambled) versions. In the scrambled landscapes, the topology (i.e. connectivity) of the landscape is preserved but the fitness values are randomly shuffled. We also compare the roughness and path predictability characteristics of the model and the experimental landscapes for 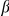-lactamase [25] and sesquiterpene synthase [28] to those for noisy additive landscapes with a continuously tunable amount of roughness.

### Evolutionary path predictability in fitness landscapes

#### Deviation from additivity, local roughness, peak fraction, and monotonic paths

**Figure 2:**
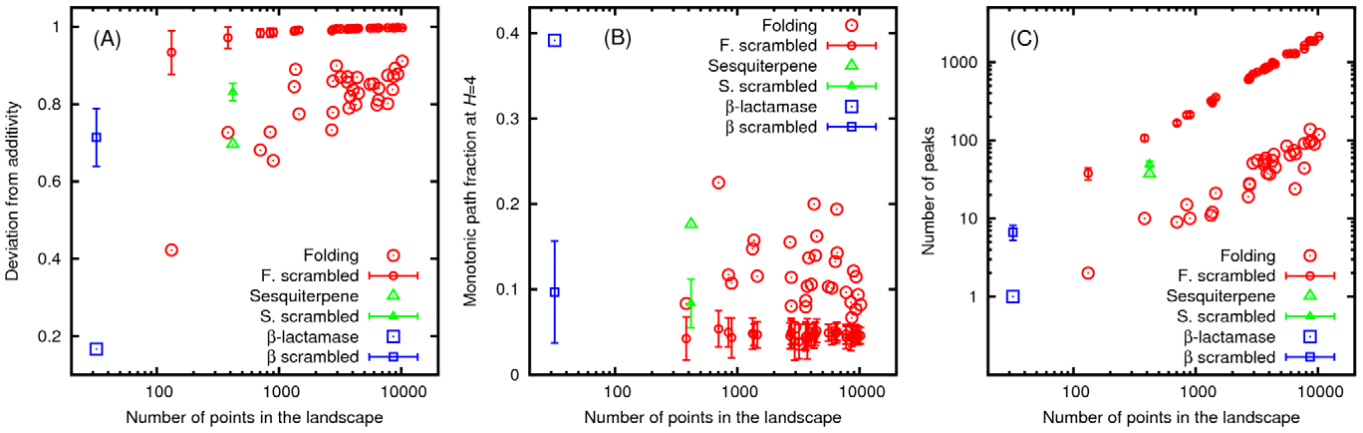
Deviation from additivity, monotonic paths and suboptimal peak suppression in folding and experimental landscapes. (A) Deviation from additivity for the folding landscapes (larger symbols), their scrambled versions (smaller symbols) and the two experimental landscapes. Error bars show one standard deviation within the ensemble of permuted landscapes. (B) Fraction of monotonic paths to the main peak in folding, scrambled and experimental landscapes. (C) The number of peaks is vastly greater in scrambled landscapes than in folding or experimental landscapes (with the exception of the sesquiterpene synthase landscape).

We first establish that the folding and the experimental landscapes are significantly different from their randomly permuted counterparts. The deviation from additivity of the folding landscapes is typically several standard deviations below the mean of their scrambled counterparts. Although the additivity hypothesis accounts for less than 40% of the fitness variability (computed by comparing the sum of the squares of the fitnesses in the landscape to the sum of the squares of the residuals of the additive fitness model fit) in all but one of the folding landscapes, the deviation from additivity of the permuted landscapes is substantially greater (Fig. 2A). The experimental landscapes follow the same pattern, in agreement with the earlier findings of Carneiro and Hartl [33]. Furthermore, both in the folding and in the experimental landscapes, the fraction of monotonic paths to the main peak is several standard deviations greater than in the respective scrambled landscapes (Fig. 2B). An even more striking disparity exists between the fraction of peaks in the folding landscapes and their permuted versions: the folding landscapes contain at least an order of magnitude fewer peaks than their scrambled counterparts; the experimental landscapes resemble the folding landscapes more closely than their own randomized versions (Fig. 2C).

**Figure 3:**
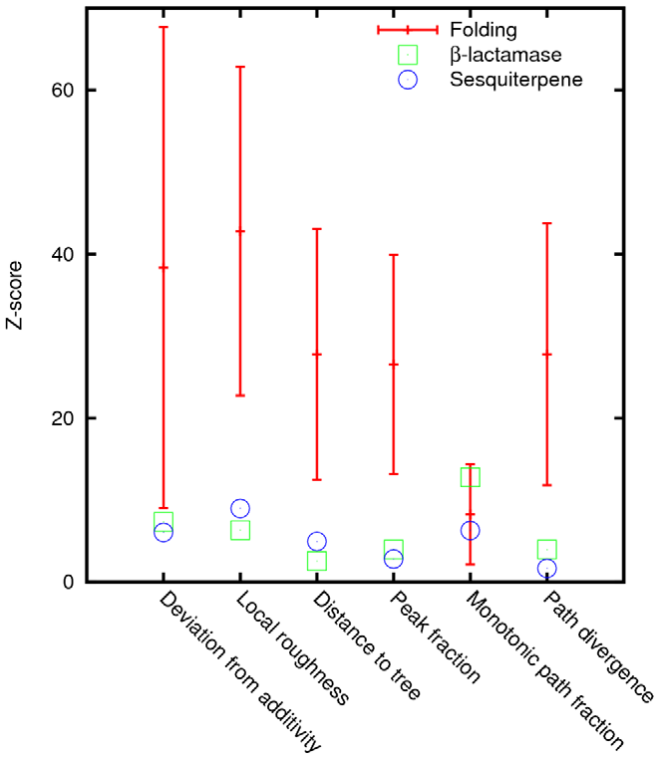
The Z-scores of different characteristics of the original folding and experimental landscapes measured with respect to the ensembles of their randomly permuted counterparts.

To further characterize the deviation of the folding and experimental landscapes from their permuted counterparts, each landscape metric was measured and the mean and standard deviation were computed among 100 randomly permuted landscapes. We then compute the Z-score (deviation from the mean measured in the units of the standard deviation) of the original non-permuted landscape compared to the ensemble of the permuted landscapes. This Z-score shows how much more correlated the original landscape is, as measured by the chosen characteristic, compared to its scrambled counterparts (Fig. 3). Notably, despite the considerable scatter of the Z-score values for the folding landscapes, they all showed extremely large difference (mean Z-score greater than 20 standard deviations) from the scrambled landscapes for all measures, with the sole exception of the monotonic path fraction (Fig. 3). The two experimental landscapes also significantly differed from the scrambled landscapes albeit less so than the folding landscapes, again with the exception of the monotonic path fraction in which case the two classes of landscapes had similar Z-scores (Fig. 3).

**Figure 4:**
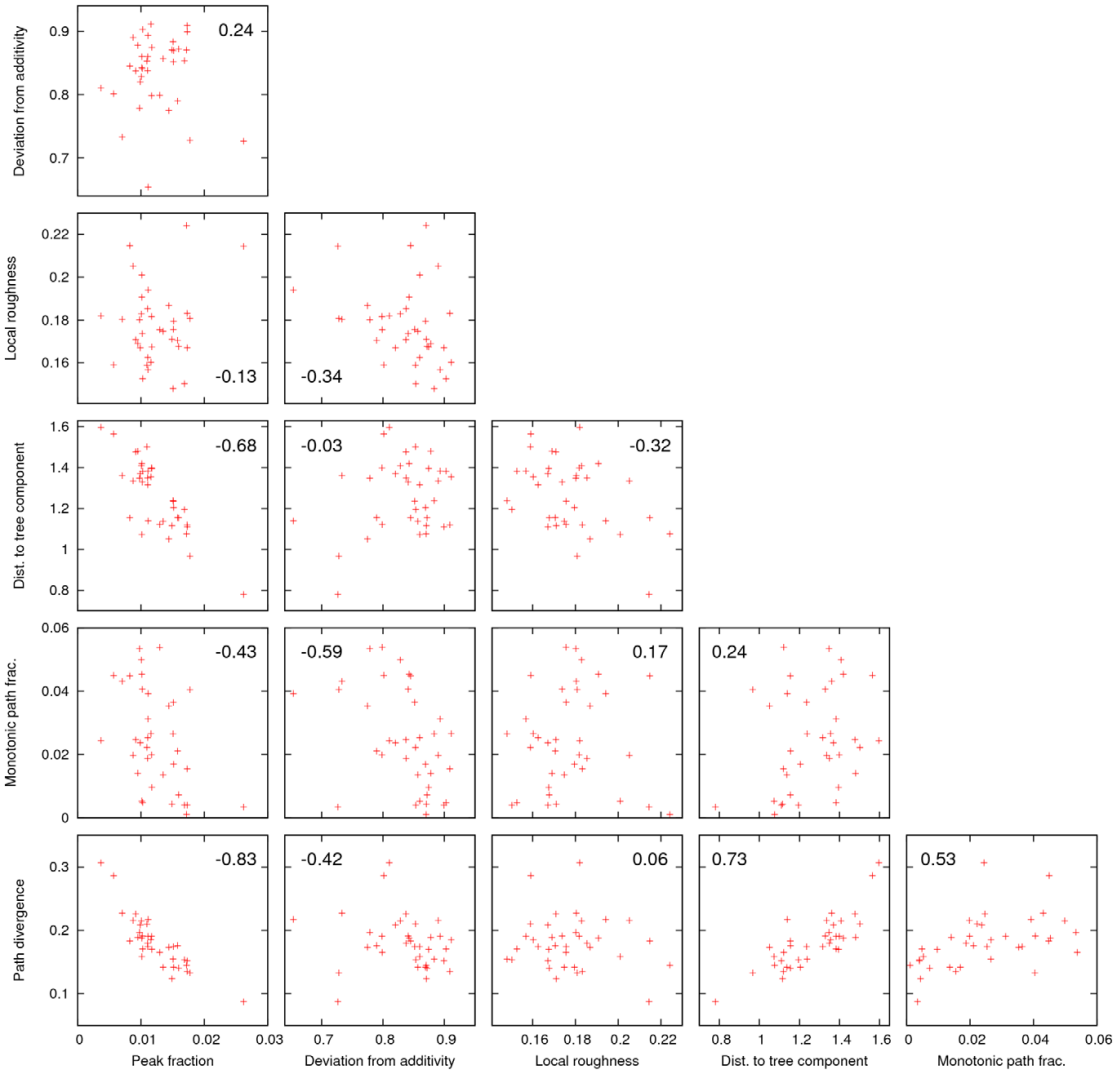
Correlations between different quantitative characteristics of the folding landscapes. Each panel quotes the Spearman rank correlation coefficient between the particular pair of characteristics.

Aside from the significant correlation (Spearman 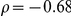) between peak fraction and mean distance to the tree component, there was little or no correlation between the four measures of landscape roughness (Fig. 4). Roughness of landscapes of high and variable dimensionality is impossible to capture by a single quantity. Therefore, the different measures seam to reveal distinct aspects of landscape architecture. The strong negative correlation between the peak fraction and mean distance to the tree component is due to the fact that each peak spawns a distinct subset of the tree component. The higher the density of peaks on the landscape, the larger fraction of the landscape that is covered by the tree component. Therefore the average distance to the tree component declines with the increasing density of peaks.

#### Path divergence

**Figure 5:**
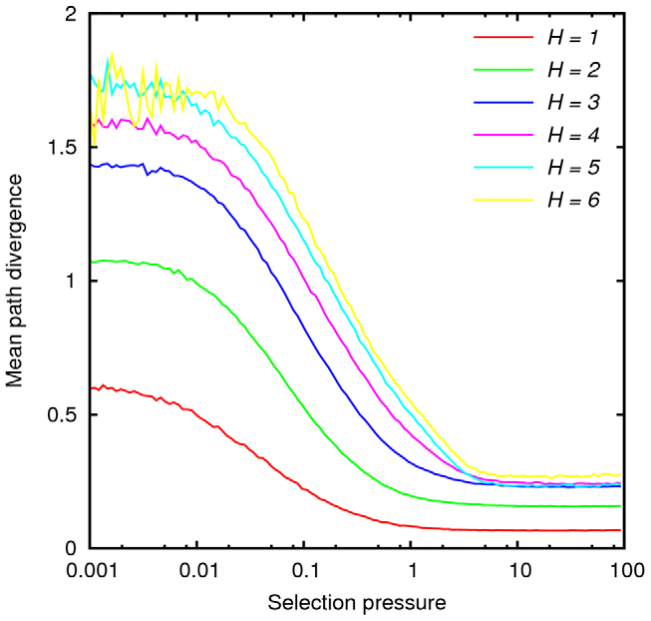
Mean path divergence as a function of selection pressure, which is a product of 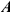 and 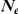, for a folding landscape with 5936 nodes and 65 peaks. Solid lines are labeled by the Hamming distance between the pairs of starting and ending points of the trajectory bundles over which the path divergence is averaged.

Starting from a random non-peak sequence in the landscape, we introduced random mutations and accepted or rejected them according to equation (3) until the trajectory arrived at a fitness peak. This procedure was repeated a large number of times, and path bundles were constructed for all pairs of starting and ending sequences. Then the mean path divergence was computed for each path bundle using equation (2) and averaged over all bundles for which starting and ending points were separated by the same Hamming distance. When selection is weak, all mutations which do not result in a sequence with zero folding probability are accepted. Thus, evolution is a random walk on the landscape and the statistical properties of evolutionary trajectories are fully determined by the topology of the landscape (i.e. the connectivity of each node). Conversely, in the strong selection limit, only mutations that increase fitness are fixed. The mean path divergence varies smoothly between the two limits (Fig. 5) and saturates at high selection pressure. In our analysis, we focus on the strong selection limit plateau. In the weak selection limit, the diversity of trajectories stems solely from the number of neighbors of each point; by contrast, in the strong selection limit, the statistics of the monotonic trajectories depend on the roughness of the landscape. Thus, the weak selection limit probes only the topology of the landscape whereas the strong selection limit also exposes its topography which appears to be critical for assessing predictability of evolution under strong selection.

#### Predictors and correlates of path divergence and monotonic path fraction

**Figure 6:**
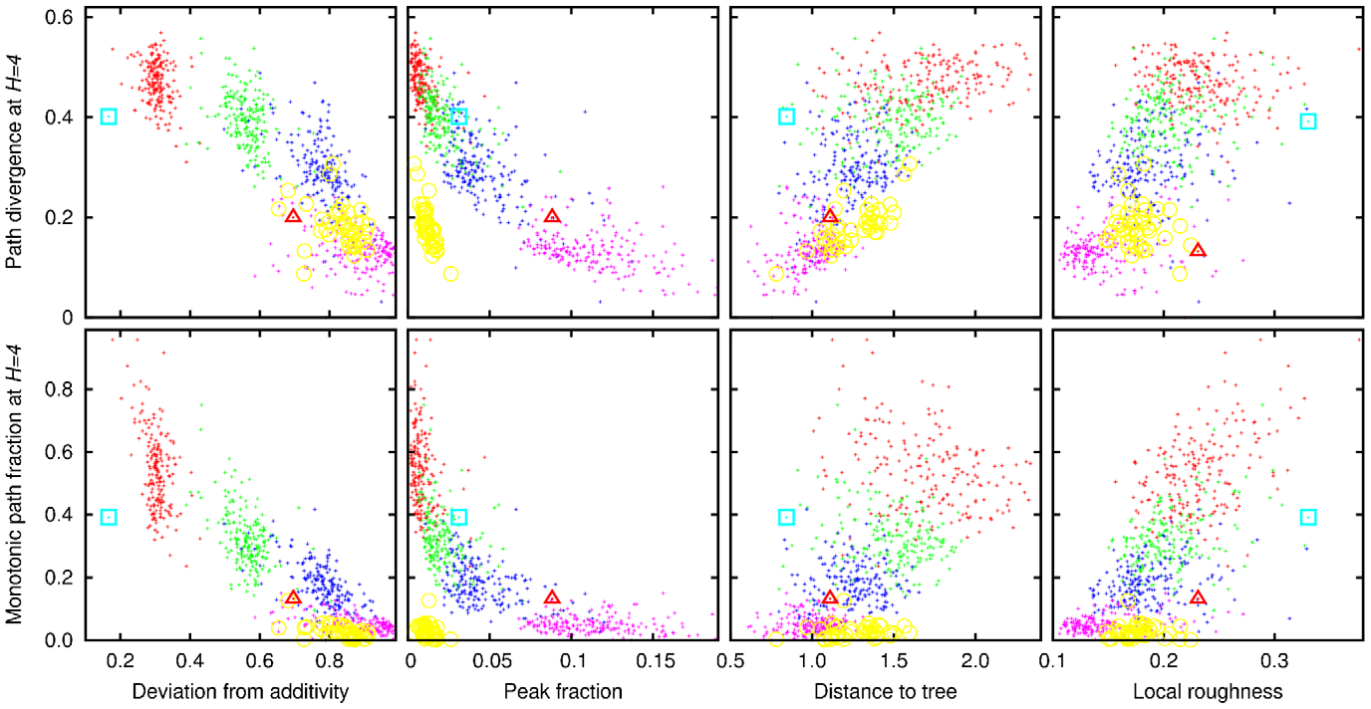
The dependence of the path divergence (top row) and the monotonic path fraction (bottom row) on the measures of landscape roughness. The dots of different color correspond to noisy additive landscapes with differing amounts of multiplicative noise: low (red), two intermediate levels (green smaller than blue), and high (magenta). Yellow circles represent the folding landscapes, the cyan squares–the 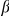-lactamase landscape, and the red triangles–the sesquiterpene synthase landscape.

**Figure 7:**
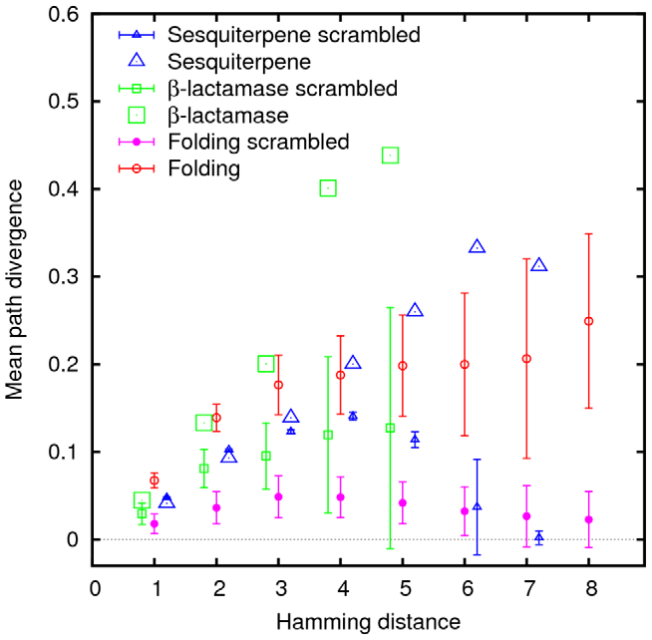
Mean path divergence in folding and experimental landscapes (larger symbols) landscapes, as well as their scrambled versions (smaller symbols) as a function of Hamming distance from the main peak.

All four measures of landscape roughness can serve as predictors of path divergence and monotonic path fraction to some degree (Fig. 6), in agreement with the notion that each of these measures reflects salient properties of fitness landscapes. The properties of the folding and empirical landscapes are consistent with those of additive landscapes that were perturbed by a moderate amount of noise (see Methods for details). A striking exception is the dearth of peaks and monotonic paths in folding landscapes all other characteristics being similar. Deviation from additivity and fraction of peaks are negatively correlated with path divergence. This relationship captures the intuitive notion that in rough landscapes there are fewer accessible evolutionary paths than in smooth landscapes, and furthermore, in rough landscapes, even those paths that are accessible show the tendency to aggregate within small areas on the landscape. Indeed, in both the folding model-derived landscapes and the experimental landscapes, the mean path divergence for all Hamming distances between the starting and ending points was dramatically greater than in scrambled landscapes (Fig. 7). Interpreting these findings in terms closer to biology, the fitness landscapes derived from the model as well as experimental landscapes show greater robustness to mutations than random landscapes: a random mutation in a model-derived or experimental fitness landscape is more likely than expected for random landscapes to have no adverse effect on the evolutionary search for greater fitness, leading to another monotonic path to the main peak. Consequently, evolution on the model-derived and experimental landscapes is less predictable (deterministic) than it would be on uncorrelated random landscapes.

In contrast to deviation from additivity, the mean distance to the tree component is positively correlated with path divergence. When the tree component comprises a large fraction of the landscape, the mean distance to the nearest tree branch is small. Consequently, the path divergence is reduced as the paths that reach the tree component do not deviate from each other from that point onward. By the same token, when the tree component is large, there are fewer monotonic paths.

The origin of the positive correlation between the local roughness and path divergence (Fig. 6) is less obvious. Paradoxically, greater noise results in lower mean local roughness of noisy additive landscapes. The lowering of the overall mean fitness with noise and, more importantly, the flattening of the mean fitness dependence on the distance from the peak (Fig. 1) appear to provide an explanation for this counter-intuitive result. Indeed we found that in noisy additive landscapes there is a characteristic fitness value of approximately 0.2 above which roughness increases with increasing noise and below which roughness declines with increasing noise. Given that roughly 75% of the points on the landscape have fitnesses below 0.2, the landscape-averaged local roughness declines with increasing noise amplitude.

## Discussion

Here we examined the fraction of monotonic paths and introduced mean path divergence as quantitative measures of the degree to which the starting and ending points determine the path of evolution on fitness landscapes. The lower the mean path divergence value, the more deterministic (and predictable) evolution is. Global measures of landscape roughness correlate with path divergence in the three analyzed classes of fitness landscapes: additive landscapes perturbed by noise, landscapes derived from our protein folding model and two small empirical landscapes. The folding landscapes are substantially smoother than their permuted counterparts. As a result, although in all analyzed landscapes only a small fraction of the theoretically possible evolutionary trajectories is accessible, this fraction is much greater in the folding and experimental landscapes than it is in randomized landscapes. In addition, the mean path divergence in the randomized landscapes is significantly smaller than in the original landscapes. Thus, the model and empirical landscapes possess similar global architectures with many more diverged monotonic paths to the high peaks than uncorrelated landscapes with the same distribution of fitness values. Consequently, evolution in fitness landscapes is substantially more robust to random mutations and less deterministic (less predictable) than expected by chance. These findings are compatible with the concept that might appear counter-intuitive but is buttressed by results of population genetic modeling, namely, that robustness of evolving biological systems promotes their evolvability [39]–[41]. Additionally, the folding landscapes exhibit a substantial deficit of peaks compared to perturbed additive landscapes and experimental landscapes, a property that translates into a substantially greater fraction of paths leading to the main peak.

When it comes to the interpretation of the properties of fitness landscapes described here, an inevitable and important question is whether the folding model employed here is sufficiently complex and realistic to yield biologically relevant information. In selecting the complexity of our folding model, we attempted to construct the simplest model which exhibits 1) a rich spectrum of low energy conformations across the sequence space, and 2) a non-trivial distribution of substitutions effects on the low energy conformations. An important choice is whether the location of monomers is confined to a lattice or can be varied continuously. When the configuration space is continuous, the distribution of energy barriers between energetically optimal conformations can extend to zero. Therefore, the subtlety of distinctions between conformations can lead to a richer structure of the fitness landscape. We chose not increase the complexity of the model further and treated monomers as point-like particles in a chain where the distance between nearest neighbors is fixed but the angle between successive links in the chain in unrestricted. Our level of abstraction is therefore somewhere between lattice models and all-atom descriptions of proteins [42]–[51].

Another important choice is the number of the model monomer types. Again, we opted for an intermediate level of abstraction and chose four types of monomers: hydrophobic, hydrophilic, and positively and negatively charged. This choice drastically reduces the size of the sequence space while retaining some of the substitution complexity whereby hydrophilic and charged monomers can be swapped under some conditions without radically altering the native state. The intermediate level of abstraction in our approach has its pros and cons. Although the model reproduces key features of protein folding such as the existence of the hydrophobic folding nucleus and two-stage folding kinetics [52], [53], compact conformations certainly do not represent proteins. Rather, we might think of our monomers as representing structurally grouped regions several (perhaps up to a dozen) amino-acids in length. Compact conformations in the model might therefore be analogous to tertiary structures of proteins. Representing sequence space with only four monomer types and treating mutations without reference to the underlying DNA or genetic code does not accurately reflect the natural mutation process. However, our goal was to isolate the features of fitness landscapes which could be traced directly to the constraints imposed by the heteropolymer folding kinetics and energetics. We therefore used a simple sequence space and a homogeneous mutation model to avoid compounding the fitness landscape structure by the complexity derived from the mutation process.

Most importantly, our folding model has been shown to reproduce the observed universal distribution of the evolutionary rates of protein-coding genes as well as the dependencies of the evolutionary rate on protein abundance and effective population sizes [34]. Therefore, despite its simplicity, the behavior of this model might reflect important aspects of protein evolution. In particular, the conclusions drawn from the analysis of the model landscapes exhaustively explored here could also apply to the fitness landscapes of protein evolution. In the previous work, we concluded that the universal distribution of evolutionary rates and other features of protein evolution follow from the fundamental physics of protein folding [34]. The results presented here suggest that the (relative) smoothness and a substantial deficit of peaks in the fitness landscapes of protein evolution that lead to mutational robustness and the ensuing evolvability could similarly follow from the fact that proteins are heteropolymers that have to fold in three dimensions to perform their functions.

The experimental landscapes considered here are decidedly incomplete. Due to experimental limitations, only the analysis of binary substitutions at a handful of sites is feasible at this time. The incompleteness of the empirical landscapes analyzed in this work could be the cause of the observed lack of peak suppression. This proposition will be put to test by the study of larger parts of experimental landscapes that are becoming increasingly available.

## Methods

### Folding model

The goal of this study is to explore the relationship between roughness and path divergence in realistic fitness landscapes. Our polymer folding model provides a simple way of constructing such landscapes. The model has been described in detail previously [34].

In brief, the model polymer is a flexible chain of monomers in which the nearest neighbors interact via a stiff harmonic spring potential with rest length 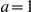. The angles between the successive links in the chain are unrestricted. There are four types of monomers: hydrophobic H, hydrophilic P, and charged + and −. Next nearest neighbors 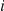 and 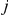 in the chain and beyond interact via a pairwise potential
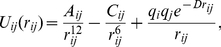
(4)where 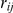 is the distance between monomers 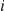 and 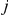, 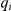 is the monomer's charge, 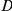 is the Debye-Hückel screening length, and 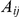 and 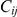 depend on the pair in question. The interaction parameters are chosen to mimic the essential features of the amino-acid interactions. To emulate the effects of solvent, we assign a stronger attraction to the HH pair than to the PP, ++, and −− pairs. There is also a long range repulsion between H and P and even stronger repulsion between H and the charged monomers. The values of the parameters are 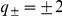, Debye-Hückel screening length 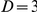. The Lennard-Jones coefficients 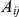 and 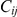 are
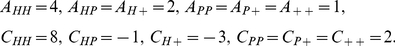
(5)Note that a 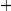 can be substituted by a 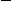 in the subscripts and the coefficients are symmetric with respect to the interchange of the indices.

The energy of the chain is
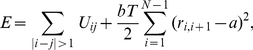
(6)where the first term is the sum of the pairwise energies given by Eq. (4) over non-nearest neighbor pairs, and the second term reflects the springs connecting nearest neighbors. The spring constant is proportional to temperature 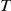. The parameters are fixed for all simulation runs at 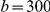, and the quench temperature 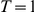. To mimic the observed tendency of the 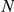 and 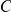 termini to be in close proximity, we fixed the endpoint monomers of the model sequences to be of 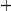 and 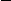 types.

Dynamics of folding are simulated via over-damped Brownian kinetics which are appropriate when inertial and hydrodynamic effects are not important. Units are chosen so that each component 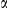 of the 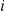
'th monomer's coordinates 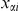 is updated according to
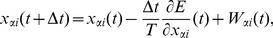
(7)where 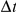 is the time step and 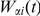 is a random variable with zero mean, variance 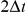, uncorrelated with 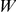 for other times, monomers and spatial directions.

### Native structure ensemble and correct folding probability

The “native structure” of a particular sequence is represented by an equilibrium ensemble of conformations. The ensemble is constructed by identifying the typical folded conformation and measuring the characteristic RMSD 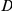 due to thermal fluctuations in the folded state. Three thousand quenches are then performed and the resulting folded conformations are accumulated. The equilibrium ensemble that represents the native structure is defined as the largest cluster of quenched conformations within RMSD distance 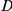 from each other. Thus, each conformation in the ensemble differs from any other by an amount comparable to the differences introduced by thermal fluctuations alone.

The concept of the native structure ensemble allows us to compute the probability that a sequence folds to a particular structure in a natural, physically plausible fashion. Given a native structure ensemble we assess its conformation space density by computing the distance 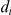 between each member 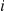 of the ensemble and its closest neighbor. Given the set 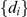 of these shortest distances we compute the median 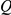 and the median absolute deviation (MAD) 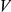. A new conformation is deemed to belong to the ensemble if the shortest distance from this conformation to the members of the ensemble is smaller than 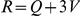.

Given a native structure ensemble of some sequence 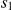 we compute the probability 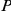 that sequence 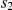 (which could be 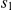 itself) folds to the this structure by accumulating 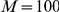 equilibrated quenched conformations of 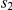 and using the above criterion to determine the fraction 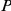 that belong to the native structure ensemble of 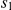. Because 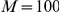 sample conformations are computed, the smallest measurable 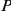 is 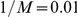. The sample size used to measure 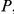 dictated by the computational demands of the model, introduces a random component to the model fitness landscapes. As we report below, model landscapes turn out to be substantially smoother than random. Therefore the underlying global structure of the model landscapes appears to survive the modest amount of randomness introduced by the relatively small sample size used for measuring 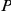.

### Search for compact robust folders

Robust folders (sequences with a high probability of correct folding) tend to have large linear regions stretched by repulsive Coulomb interactions. Because the linear regions have no contacts with other monomers, we focused our attention on compact conformations with a high monomer contact density. Substitutions in these higher complexity conformations were more likely to exhibit non-trivial effects. To find compact robust folders in the vast available sequence space of 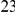-mers (the sequences are of length 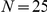 but the endpoint monomer types are fixed) with 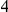 monomer types, we implemented a simulated annealing search which optimized the correct folding probability 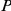 divided by the cube of the native conformation's radius of gyration. The search produced over 800 sequences with 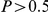 and at least two distinct regions of the polymer in mutual contact.

### Assembly of the folding fitness landscapes

We examined each single substitution mutant of a robustly folding sequence and computed the folding probability 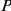 to the structure of the original sequence. All mutants with 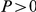 were added to the landscape and if 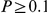 their mutants were also examined. This process is repeated until all mutants of the last sequence under consideration have 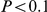.

From our study of complete landscapes we estimate that on average for each sequence with 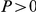 which is included into the landscape, roughly 6 others with 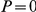 need to be examined. Since each quench and equilibration takes about 2–4 seconds, landscape construction takes roughly 30 minutes to an hour per included sequence. Thus landscapes larger than 10,000 sequences take months to compile.

At the time of submission, 39 complete landscapes have been constructed, the largest comprising 12969 sequences.

### Additive landscapes perturbed by noise

The organization of the folding fitness landscapes and experimental landscapes were compared with perfectly additive landscapes perturbed by noise constructed as follows. Each substitution to the peak fitness sequence was assigned a negative fitness differential drawn at random from an exponential distribution with parameter 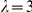. The sum over the fitness differentials of a particular set of substitution was modified by either additive of multiplicative noise [54]. Additive noise is drawn from a Gaussian distribution with zero mean and standard deviation 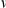 which was varied between 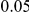 and 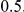 The multiplicative perturbation is achieved by multiplying the fitness by a number drawn from a uniform distribution 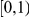 raised to a positive power 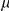 varied between 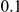 and 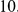 When 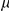 is small, multiplicative factors are close to unity and the perturbation is small as well. If the perturbed fitness was positive, the mutant was included into the landscape. The noise amplitude was varied to obtain a family of landscapes of continuously varying roughness. Only the data for the additive landscapes with multiplicative noise were included in this manuscript. Landscapes perturbed by other types of noise exhibited essentially the same qualitative behavior.

### Experimental landscapes

The studies on experimental fitness landscapes typically involve constructing a library of all possible combinations of binary mutations at a small number of sites. The first study included in the present analysis measured the minimum inhibitory concentrations (MIC) of an antibiotic for a complete spectrum of mutants with modified TEM 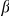-lactamases; the transition from the antibiotic-sensitive to the antibiotic-resistant form requires five mutation, so the landscape encompassed 120 mutational trajectories between the most distant points on the landscape (or 32 sequences) [25]. The logarithm of MIC was used as the proxy for fitness. In the second study, catalytic activity of 419 sesquiterpene synthase mutants that differed by at most 9 substitutions was measured [28]. We used the catalytic specificity (propensity for producing a particular reaction product rather than a broad spectrum of products) of the mutant enzymes as the proxy for fitness. Before performing the analysis, the fitnesses in the experimental landscapes are mapped onto the 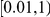 interval to enable meaningful quantitative comparisons of the roughness measures.
